# Dr. Carlos John Finley

**Published:** 1915-10

**Authors:** 


**Dr. Carlos John Finley,** noted for his work in yellow fever, died in Havana, Cuba, aged 81, August 20.

